# The Prognostic Impact of the Ki-67 Proliferation Index in Patients with Surgically Treated Spinal Metastases

**DOI:** 10.3390/cancers18081210

**Published:** 2026-04-10

**Authors:** Saif-Eldin Abedellatif, Marija Janjic, Logman Khalafov, Harun Asoglu, Juliane Dittmer, Muriel Heimann, Mohammed Jaber, Haitham Alenezi, Marieta Ioana Toma, Matthias Schneider, Hartmut Vatter, Motaz Hamed, Mohammed Banat

**Affiliations:** 1 Department of Neurosurgery, University Hospital Bonn, University of Bonn, Venusberg Campus 1, Building 81, 53127 Bonn, Germany; 2 Institute of Pathology, University Hospital Bonn, 53127 Bonn, Germany

**Keywords:** spinal metastasis, Ki-67 index, biomarker, surgical treatment, overall survival

## Abstract

Background: The prognostic assessment of patients with spinal metastases is primarily based on clinical and radiological parameters. Biological tumor characteristics such as the proliferation marker Ki-67 have prognostic relevance in various metastatic settings. This study aimed to evaluate the prognostic impact of the Ki-67 proliferation index on survival outcomes in patients undergoing surgery for spinal metastases. Methods: We included 166 patients who underwent surgical treatment for spinal metastases at our university clinic between 2015 and 2024. Clinical, functional, tumor-related, and perioperative variables were collected. Receiver operating characteristic (ROC) analysis was performed to evaluate the discriminatory ability of Ki-67, and comparisons were made between patient groups according to Ki-67 expression (≤20% vs. >20%). Results: Based on ROC analysis, Ki-67 demonstrated a moderate but significant predictive ability for 1-year mortality (area under the curve [AUC]: 0.69, *p* = 0.001). Patients with a Ki-67 index of >20% showed a significantly shorter overall survival than those with a lower Ki-67 index of ≤20% (median overall survival: 5.0 vs. 14.5 months, *p* < 0.001). One-year mortality was significantly higher in the high Ki-67 group (78.9% vs. 41.8%, *p* = 0.001). High Ki-67 expression was associated with more aggressive tumor characteristics but was not associated with increased perioperative morbidity. Conclusions: The Ki-67 proliferation index is a significant prognostic biomarker in surgically treated patients with spinal metastases. A Ki-67 index threshold of 20% identifies patients at increased risk of early mortality and significantly reduced overall survival.

## Simple Summary

Survival in patients with spinal metastases varies widely, making reliable prognostic tools essential for treatment decisions. Beyond established clinical and imaging-based factors, tumor biology may provide additional insight. This study evaluated whether the proliferation marker Ki-67 can help predict survival in patients undergoing surgery for spinal metastases. Higher Ki-67 levels were associated with a worse prognosis, including shorter overall survival (5.0 vs. 14.5 months) and higher 1-year mortality. Despite its association with more aggressive tumor characteristics, elevated Ki-67 was not linked to increased perioperative complications. These findings suggest that Ki-67 is a useful biomarker for identifying high-risk patients and may support more individualized treatment planning.

## 1. Introduction

In recent decades, metastases affecting the spinal column have assumed substantial epidemiological relevance [1,2]. Clinically evident spinal metastases occur in approximately 10–20% of patients with solid tumors, with the spine being the most common site of osseous dissemination. The most frequent primary tumors are breast, prostate and lung cancers, followed by renal, gastrointestinal and hematologic malignancies [3,4,5,6].

Improvements in standards of care and targeted systemic therapies have markedly extended patient survival, necessitating a reassessment of treatment strategies. Surgery has therefore emerged as an essential component of contemporary multidisciplinary management of spinal metastases, particularly in selected patients with neurological compromise or mechanical instability [7,8,9,10].

Despite substantial advances in surgical and systemic therapies, prognosis in metastatic spinal disease remains markedly heterogeneous and is modulated by tumor biology, overall disease burden, and neurological status. To support treatment selection, several prognostic and decision-support models, most notably the Tokuhashi and Tomita scores for survival estimation and the spinal instability neoplastic score (SINS) for mechanical assessment, have been developed and are now widely implemented in clinical practice [11,12]. However, these tools rely predominantly on clinical variables and radiological features and thus provide only indirect insight into the biological aggressiveness of metastatic disease, which limits their ability to fully explain the observed variability in individual outcomes fully. However, outcomes remain heterogeneous even within the same prognostic strata, suggesting that conventional models do not adequately capture the underlying biological aggressiveness of metastatic disease.

Ki-67 is a non-histone nuclear protein first described by Gerdes and colleagues in 1983 and is expressed exclusively in proliferating cells during the G1, S, G2 and M phases of the cell cycle [13,14,15]. The Ki-67 proliferation index is therefore widely used as an immunohistochemical marker of cellular proliferation and has demonstrated robust prognostic relevance across numerous primary tumor entities and metastatic sites, with higher expression consistently associated with more aggressive tumor behavior and reduced survival [16,17,18,19,20]. Nevertheless, the prognostic impact of Ki-67 in the specific context of surgically treated spinal metastases has not been systematically defined, and it remains unclear whether integrating this biological marker into preoperative risk stratification provides incremental value over established clinical and radiological factors. The present study therefore aims to evaluate the prognostic impact of the Ki-67 proliferation index on survival outcomes in patients undergoing surgery for spinal metastases, with the goal of refining risk stratification and informing individualized post-surgical decision making.

## 2. Methods

### 2.1. Patients and Inclusion Criteria

This retrospective cohort study identified 200 patients who underwent surgical treatment for spinal metastases between 2015 and 2024. 166 patients fulfilled our inclusion criteria und were included. The criteria for inclusion in this study were known histopathologically confirmed spinal metastases in all spinal column localizations, an age greater than 18 years, the availability of the Ki-67 index, and treatment via neurosurgical resection.

### 2.2. Data Recording

Comprehensive clinical and neurological data were collected, including patient age, sex, comorbidities, primary tumor type, spinal metastases location, details of the neurosurgical procedure, extent of vertebral involvement, and preoperative risk status according to the American Society of Anesthesiologists (ASA) classification. Clinical and neurological functions were assessed using the American Spinal Injury Association (ASIA) impairment scale.

Functional performance on admission was additionally evaluated using the Karnofsky Performance Scale (KPS), dichotomized into KPS ≥ 70% and KPS < 70% as previously described [21]. The Charlson Comorbidity Index (CCI) was used to quantify the preoperative comorbidity burden.

To identify spinal surgery-related complications, postoperative records were systematically screened for cerebrospinal fluid leakage, postoperative meningitis, implant failure, and new or aggravated neurological deficits. Perioperative complications were defined as any postoperative adverse event, with or without surgical revision, occurring within 30 days after the index procedure.

Overall survival was calculated from the date of spinal metastasis resection to death, following established methodology [21,22]. Patients for whom follow-up survival data were unavailable, typically due to continued treatment at external institutions, were excluded from our survival analysis.

### 2.3. Patient Groups

Patients were retrospectively classified into two groups according to their Ki-67 index: low proliferative (≤20%) and high proliferative (>20%). The optimal cut-off value for the Ki-67 index was determined using receiver operating characteristic (ROC) analysis for the prediction of 1-year mortality, yielding an optimal threshold of approximately 22–23%. Since Ki-67 values in pathology reports were documented in discrete increments (e.g., 5%, 10%, 15%, 20%, 25%) and no exact values within this range were available, a clinically practical cut-off value of 20% was selected for stratification.

### 2.4. Study Design

This study adhered to the ethical principles outlined in the 1964 Helsinki Declaration and received approval from the local ethics committee of the University Hospital Bonn (reference no. 067/21) on 19 November 2021. Given the retrospective nature of the study, the acquisition of informed consent from participants was not pursued.

### 2.5. Histopathology

All surgically resected spinal metastasis specimens were evaluated using hematoxylin and eosin staining and subjected to immunohistochemical analysis. Depending on the presumed primary tumor origin, established diagnostic antibodies were applied for tumor classification. In addition, immunohistochemical staining for Ki-67 was performed using a monoclonal antibody (Clone Ki-67P, dilution 1:1000, Dianova, Hamburg, Germany). The Ki-67 proliferation index was assessed in randomly selected high-power fields by determining the proportion of Ki-67-positive nuclei relative to the total number of tumor cell nuclei and was expressed as a percentage.

### 2.6. Exclusion Criteria

We excluded all patients without known Ki-67 proliferation or with incomplete data. Furthermore, we excluded patients who were not operated on.

### 2.7. Statistical Analysis and Graphical Illustrations

Data were analyzed using IBM^®^ SPSS^®^ Statistics for Mac (version 31.0; IBM Corp., Armonk, NY, USA). Receiver operating characteristic (ROC) curves were constructed to assess the ability of the Ki-67 proliferation index to predict 1-year mortality, and the optimal cut-off value was determined using the Youden Index. Baseline demographic, clinical, tumor-related, surgical, and postoperative variables were compared between patients with a low (≤20%) and high (>20%) Ki-67 index. Categorical variables were analyzed using Pearson’s χ^2^ test or Fisher’s exact test, and continuous variables were analyzed using the independent samples *t*-test or Mann–Whitney U test, as appropriate. A multivariable binary logistic regression analysis was performed to identify independent predictors of a high Ki-67 index (>20%). Dichotomized variables were assessed using the Wald test, and results are presented as adjusted odds ratios (aORs) with 95% confidence intervals. Statistically significant variables were incorporated into a preoperative clinical scoring system, whose discriminatory ability was evaluated by ROC analysis. Overall survival was analyzed using the Kaplan–Meier method and compared using the log-rank test. To assess the independent prognostic impact of Ki-67 on overall survival, a multivariable Cox proportional hazards regression analysis was performed, including clinically relevant variables. Results are reported as hazard ratios (HRs) with 95% confidence intervals. All tests were two-sided, and a *p*-value < 0.05 was considered statistically significant. Graphical illustrations were generated using GraphPad Prism (version 10.0; GraphPad Software, San Diego, CA, USA).

## 3. Results

### 3.1. Patient Characteristics

**Figure 1:**
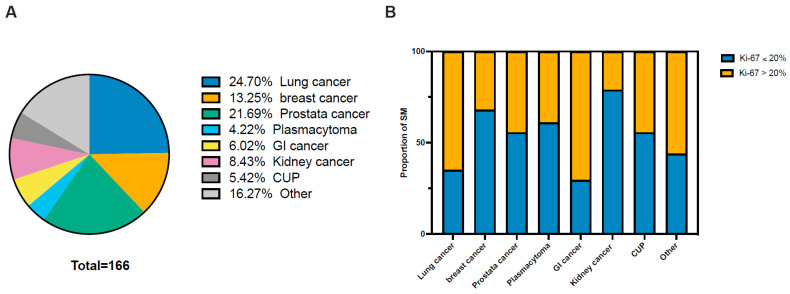
Patient cohort characteristics and Ki-67 expression in spinal metastases. (**A**) Distribution of primary tumor origins across all surgically treated spinal metastases in the study cohort. (**B**) Proportion of spinal metastases with Ki-67 ≤ 20% and >20% stratified by primary tumor entity.

Between 2015 and 2024, a total of 200 patients underwent neurosurgical treatment for spinal metastases at the Department of Neurosurgery, University Hospital Bonn. Of them, 166 patients fulfilled the inclusion criteria with complete data. The cohort comprised 99 male (59.6%) and 67 female (40.4%) patients. The most frequent primary tumor origin was the lung (24.7%), followed by the prostate (21.7%) and breast (13.3%) (Figure 1A). Ki-67 expression levels (≤20% vs. >20%) in spinal metastases vary according to the primary tumor entity (Figure 1B). Extraspinal metastatic dissemination was observed in 56.0% of patients. The median overall survival for the entire cohort was 8.55 months (interquartile range [IQR]: 3–17 months), with a 1-year mortality rate of 51.8%. Detailed patient and tumor-specific characteristics are presented in Table 1.

### 3.2. Patient-Related and Disease-Related Factors Associated with Ki-67 Index

The further analysis found that 102 patients (61.4%) exhibited a low Ki-67 index (≤20%), whereas 64 (38.6%) had a high Ki-67 index (>20%). Baseline demographic characteristics, including sex and age at spinal metastasis diagnosis, did not differ significantly between the two groups. Patients with a high Ki-67 index presented with a significantly poorer preoperative general condition, as reflected by a higher proportion of ASA scores > 2, than patients with a low Ki-67 index (73.4% vs. 57.8%, *p* = 0.04).

With respect to primary tumor entities, spinal metastasis from lung cancer was significantly more frequent in patients with a high Ki-67 index than in those with a low Ki-67 index (34.4% vs. 18.6%, *p* = 0.022), whereas the distribution of other primary tumor types did not differ significantly between groups. The time from primary tumor diagnosis to spinal metastasis diagnosis (time to progression—TTP) was significantly shorter in patients with a high Ki-67 index, with a median of 0 years (IQR 0–1.5) compared to 1 year (IQR 0–8) in the low Ki-67 group (*p* = 0.012). Consistently, spinal metastases occurring within 1 year of primary tumor diagnosis were significantly more common in the high Ki-67 group (57.4% vs. 43.8%, *p* = 0.019). Furthermore, spinal metastases as the first manifestation of metastatic disease occurred more frequently in patients with a high Ki-67 index (synchronous metastases: 55.7% vs. 39.6%, *p* = 0.048).

No significant differences were observed between the two groups regarding spinal metastasis location, number of spinal segments involved, presence of spinal cord compression, spinal instability, or systemic metastatic burden, including the presence, number, and localization of extraspinal metastases. Preoperative neurological and functional status, assessed by ASIA grade and Karnofsky Performance Scale, were comparable between groups.

Surgical treatment characteristics, including type of surgery, operative time, and intraoperative blood loss, did not differ significantly between patients with low and high Ki-67 indices. Likewise, postoperative complication rates, revision surgery, neurological outcomes, functional recovery, local tumor recurrence, and readmission rates showed no significant group differences. Additional clinical, tumor, and other characteristics in patients with a low or high Ki-67 index are detailed in Table 2.

### 3.3. Prognostic Value of the Ki-67 Index for 1-Year Mortality and Overall Survival

**Figure 2:**
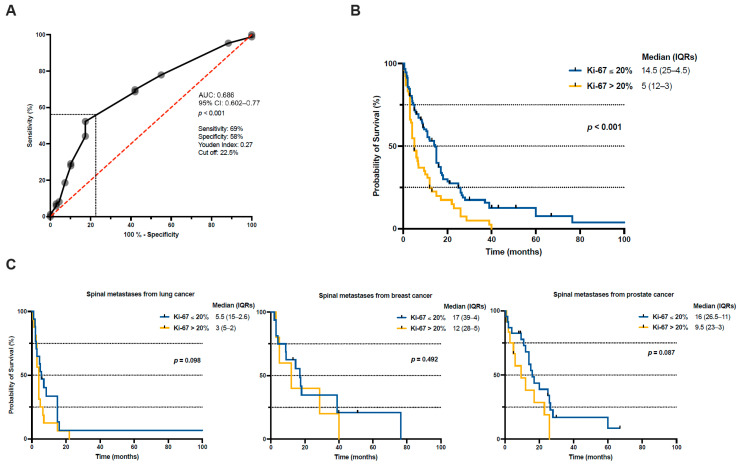
(**A**) ROC curve illustrating the performance of the Ki-67 proliferation index for predicting 1-year mortality after surgery for spinal metastases. (**B**) Kaplan–Meier analysis showing significantly shorter overall survival in patients with Ki-67 > 20% than those with Ki-67 ≤ 20% in the overall cohort. (**C**) Subgroup Kaplan–Meier analyses stratified by primary tumor origin (lung, breast and prostate cancer). AUC, area under the curve; CI, confidence interval; IQR, interquartile range; ROC, receiver operating characteristic.

The prognostic value of the Ki-67 proliferation index for 1-year mortality was analyzed using ROC methodology. The ROC curve indicated that Ki-67 had a moderate discriminatory capacity in predicting 1-year mortality among patients undergoing surgery for spinal metastases, yielding an area under the curve (AUC) of 0.686 (95% confidence interval [CI] 0.602–0.770; *p* = 0.001) (Figure 2A). Based on the Youden Index (0.27), an optimal Ki-67 cut-off value of 22.5% was determined, corresponding to a sensitivity of 69% and a specificity of 58% for predicting 1-year mortality.

To further investigate the prognostic relevance of tumor proliferative activity, Kaplan–Meier survival analyses were conducted using a dichotomized Ki-67 threshold of 20%. Patients with a high Ki-67 index (>20%) demonstrated significantly reduced overall survival compared to those with a lower Ki-67 index (≤20%) (log-rank test, *p* = 0.001) (Figure 2B). The survival curves diverged early after surgery and remained clearly separated over time, underlining the enduring adverse prognostic impact of elevated proliferative activity. Subgroup analyses stratified by primary tumor origin showed a trend toward shorter overall survival in patients with higher Ki-67 expression across spinal metastases from prostate, lung and breast cancer; however, these differences were not statistically significant within individual tumor entities (Figure 2C).

Importantly, in multivariable Cox regression analysis, high Ki-67 expression (≥20%) remained independently associated with worse overall survival (HR: 1.98, 95% CI: 1.32–2.98, *p* = 0.001). In addition, poor preoperative performance status (KPS < 70%) (HR: 3.73, 95% CI: 1.78–7.85, *p* = 0.001) and prior oncological treatment (HR: 1.92, 95% CI: 1.12–3.27, *p* = 0.017) were identified as independent predictors of reduced survival (Table S1).

### 3.4. Multivariate Analysis of Factors Associated with a High Ki-67 Index (>20%)

**Figure 3:**
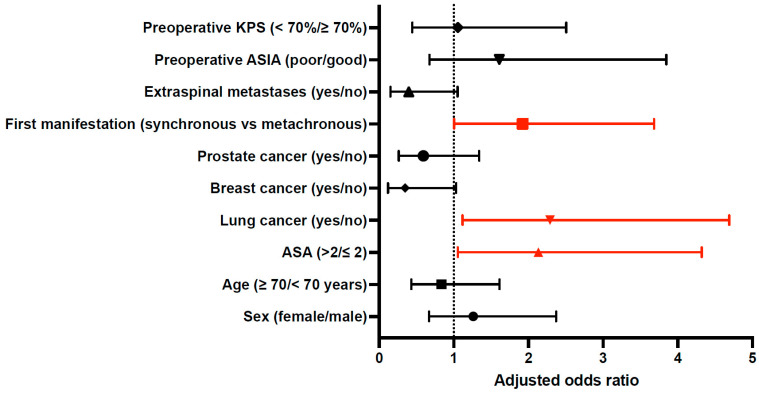
Forest plot of factors associated with a high Ki-67 index (>20%). Adjusted odds ratios with 95% confidence intervals derived from multivariate binary logistic regression are shown. The vertical dashed line indicates an odds ratio of 1 (no effect). Variables highlighted in red indicate statistically significant associations (*p* < 0.05). ASA, American Society of Anesthesiology; ASIA: American Spinal Injury Association; KPS: Karnofsky Performance Scale.

In the multivariate binary logistic regression model, several variables were independently associated with an elevated Ki-67 index (>20%) (Table 3, Figure 3). Poor preoperative physical status, indicated by an ASA score > 2, was significantly associated with a high Ki-67 index (aOR: 2.13, 95% CI: 1.05–4.32; *p* = 0.035). Similarly, lung cancer as the primary tumor entity emerged as an independent predictor of a high Ki-67 index (aOR: 2.29, 95% CI: 1.12–4.69; *p* = 0.024), whereas kidney cancer as the primary tumor type was inversely associated with a high Ki-67 index (aOR: 0.20, 95% CI: 0.04–0.94; *p* = 0.041).

Moreover, spinal metastases presenting as the initial manifestation of systemic disease (synchronous metastases) were independently correlated with a high Ki-67 index compared to metachronous lesions (aOR: 1.92, 95% CI: 1.00–3.68; *p* = 0.049). No significant associations were observed with patient age, sex, comorbidity burden, tumor extent, neurological function, radiological characteristics, intraoperative parameters, or postoperative oncological treatment modalities.

### 3.5. Preoperative Risk Score for Elevated Ki-67 Expression and Survival Stratification

**Figure 4:**
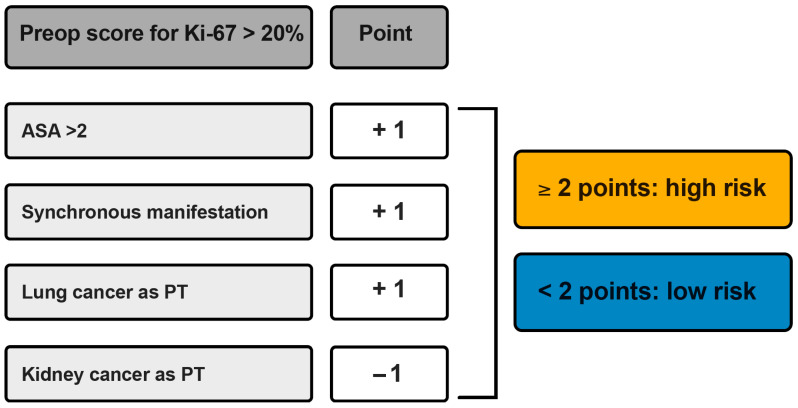
A clinical scoring system to preoperatively estimate the risk of a high Ki-67 index (>20%). ASA, American Society of Anesthesiology; PT: primary tumor.

**Figure 5:**
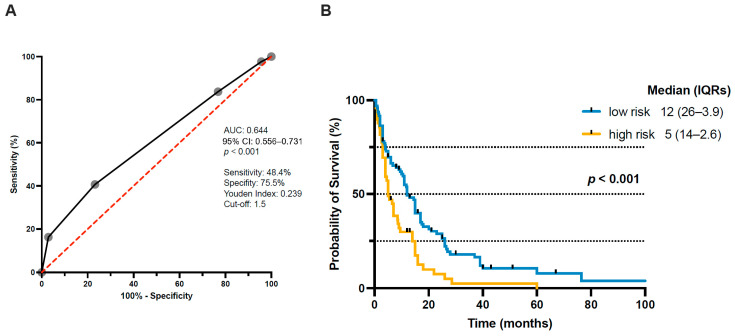
(**A**) ROC curve demonstrating the discriminatory ability of the preoperative score for detecting elevated Ki-67 expression (>20%). (**B**) Kaplan–Meier analysis of overall survival stratified by the preoperative score, with patients categorized as being low risk (<2 points) or high risk (≥2 points) for a high Ki-67 index. AUC, area under the curve; CI, confidence interval; IQR, interquartile range; ROC, receiver operating characteristic.

Based on the independent predictors identified in the multivariate logistic regression analysis, a preoperative clinical scoring system was established to estimate the likelihood of an elevated Ki-67 index (>20%) (Figure 4). The score included an ASA classification > 2, synchronous presentation of spinal metastases, and lung cancer as the primary tumor entity (each contributing +1 point), whereas kidney cancer as the primary tumor was assigned −1 point. ROC analysis demonstrated a moderate discriminatory capacity of the model for predicting elevated Ki-67 expression, with an AUC of 0.644 (95% CI 0.556–0.731) (Figure 5A). An optimal threshold of 1.5 points was determined, allowing patient stratification into a low-risk group (<2 points) and a high-risk group (≥2 points).

Kaplan–Meier survival analysis revealed a significant separation in overall survival between these risk categories. Patients classified as high risk (≥2 points) had a markedly reduced median overall survival of 5 months compared to 12 months in the low-risk cohort (log-rank *p* < 0.001) (Figure 5B). These findings suggest that the proposed preoperative clinical score not only predicts elevated proliferative activity but also serves as a practical tool for preoperative survival stratification in patients undergoing surgery for spinal metastases.

## 4. Discussion

The detection of spinal metastases during the course of a tumor disease poses a further challenge to the treatment of the underlying disease [23,24,25]. This present study analyzes the prognostic impact of the Ki-67 index in patients after neurosurgical therapy for spinal metastases. We found that a high proliferation of Ki-67 was significantly correlated with high mortality rates and poor overall survival. The spine research group at our spinal and neuro-oncology center focuses on parameters that have a significant positive or negative impact on survival in patients with spinal metastases [26,27,28]. To our knowledge, this study is the first of its kind to address this topic and systematically investigate the correlation between Ki-67 and survival. The proliferation rate of Ki-67 plays a central role in the world of oncology [13,14]. The significance of Ki-67 is evident in therapy and as a prognostic factor in the further course of treatment [17,18,19,20]. The value of Ki-67 in spinal metastases has been little studied, but other studies have shown that the proliferation rate of Ki-67 from different systemic tumor entities or bone tumor is associated with a poor outcome [29,30,31]. In our cohort, synchronous spinal metastases were significantly associated with higher Ki-67 expression than metachronous lesions. To the best of our knowledge, no previous studies have specifically examined the relationship between the timing of spinal metastatic manifestation and proliferative activity as quantified by Ki-67. A plausible explanation for this observation is that synchronous metastases may represent a biologically more aggressive disease phenotype with a greater systemic tumor burden at the time of initial diagnosis, thereby exhibiting increased cellular proliferation. It might also be hypothesized that prior systemic therapy, particularly chemotherapy, could modulate proliferative activity and consequently affect Ki-67 expression levels in spinal metastases. However, this hypothesis could not be tested in our cohort due to the limited sample size and the heterogeneity of preoperative treatment regimens. Moreover, no significant differences in recurrence rates were observed between the two groups in our cohort. Similarly, the anatomical level of spinal involvement showed no significant association with Ki-67 expression. The literature makes special mention of Ki-67 in certain tumors such as malignant melanoma, lung cancer, breast cancer, and occasionally prostate cancer [32,33,34,35]. In our series, we found a higher incidence of lung cancer with clear expression of Ki-67 whereas the accumulation was normal in the other spinal metastases of different tumor entities. As a specialized surgical center, we sought to determine whether retrospective assessment of Ki-67 influenced our findings, particularly in relation to surgically relevant factors or postoperative complication rates. However, no statistically significant differences were observed between the two groups in this regard. Importantly, a high Ki-67 proliferation index was significantly associated with poorer overall survival and increased mortality in our cohort. This observation aligns with previous evidence demonstrating that elevated proliferative activity correlates with unfavorable outcomes in patients with advanced disease, especially in the presence of extraspinal metastases or tumor recurrence [36,37,38]. High Ki-67 expression does not only have negative aspects; some authors respond to the high proliferation of Ki-67 by administering appropriate chemotherapy, thereby improving overall survival [39,40,41]. The main problem with Ki-67 detection is that the value is only available postoperatively, which can influence the prognosis. New methods for direct, rapid, intraoperative detection of Ki-67 expression may influence the surgical procedure and the associated prognostic therapeutic effect because, for example, the tumor may be resected more aggressively [42]. The expression of Ki-67 also appears to have different values depending on where the tumor occurs. Based on the ROC analysis, we have taken the value of 20% as statistically significant. In other studies—referring to systemic rather than spinal metastases—different values of 8% are shown to be significant [19,36]. In future studies, prognostic assessment in spinal metastases should move beyond isolated evaluation of the Ki-67 proliferation index. A more comprehensive biological characterization that accounts for entity-specific tumor biology is warranted, as different primary malignancies are driven by distinct immunohistochemical receptor constellations and molecular alterations. Incorporating additional biomarkers, such as receptor expression patterns (e.g., EGFR, HER2) quantified by standardized scoring systems like H-scores, as well as mutation profiling, may yield deeper insights into tumor aggressiveness, therapeutic vulnerabilities, and biological heterogeneity [31]. Moreover, the integration of systemic inflammatory biomarkers, including cytokines and interleukin-based signatures, could further refine prognostic stratification by capturing the interaction between tumor biology and the host inflammatory response. Such a multimodal, entity-specific biomarker strategy has the potential to enhance prognostic accuracy and to support more individualized treatment decision making in patients with spinal metastases. However, validation of these concepts will require large, well-annotated patient cohorts, ideally within multicenter study frameworks, to ensure adequate statistical power and generalizability across different primary tumor entities and treatment settings.

## 5. Conclusions

The Ki-67 proliferation index is a significant prognostic biomarker in surgically treated patients with spinal metastases. A Ki-67 threshold of 20% identifies patients at increased risk of early mortality and significantly reduced overall survival. The results should be considered in future stratification of patient cohorts, particularly when choosing treatment following surgical resection of spinal metastases.

## 6. Limitations

The present study has several limitations. Patients were not randomized. The surgical treatment was decided by the neurosurgeon. Acquisition of data was retrospective. Our data are therefore subject to well-known and well-described types of bias. The number of patients with Ki-67 is quite small, which means the univariate and multivariate analyses may be subject to error. The study population comprised a heterogeneous group of primary tumor entities. Given that the biological and prognostic relevance of Ki-67 may vary across different tumor types, this heterogeneity may limit the generalizability of the findings. Furthermore, the proposed preoperative score was developed and evaluated within the same cohort, which introduces a potential risk of overfitting. In addition, its discriminative performance was only moderate. Therefore, the score should be considered exploratory and requires external validation in independent patient cohorts before clinical application. Importantly, the present study primarily focuses on outcomes following surgical treatment of spinal metastases, where detailed oncological follow-up data were limited. In particular, information on systemic therapies such as chemotherapy, immunotherapy, or targeted treatments was not comprehensively available. As these factors may substantially influence survival outcomes and interact with tumor biology, including Ki-67 expression, their absence represents a relevant limitation. Future studies incorporating detailed oncological treatment variables are warranted to better elucidate the interplay between tumor proliferation, systemic therapy, and survival.

Finally, prospective multicenter studies with larger and more homogeneous patient populations are needed to validate these findings and to further clarify the role of Ki-67 in prognostic stratification of patients with spinal metastases.

## Figures and Tables

**Table 1:** Patient characteristics (*n* = 166).

	*n* = 166		*n* = 166
Gender, *n* (%)		Spinal cord compression, *n* (%) Yes No Involved segments, *n* (%) ≤2 segments ≥3 segments Extraspinal metastases, *n* (%) Yes No Postoperative ASIA, *n* (%) Good (D, E) Poor (A, B, C) Postoperative KPS, *n* (%) ≥70% <70 Overall survival, months Median (IQR) 1-year mortality, *n* (%) Yes No	147 (88.6) 19 (11.4) 86 (51.8) 80 (48.2) 93 (56) 73 (44) 113 (68.1) 53 (31.9) 103 (62) 63 (38) 8.55 (3–17) 86 (51.8) 69 (41.6)
Male	99 (59.6)		
Female	67 (40.4)		
Age at SM diagnosis, *n* (%)			
<70 years	92 (55.4)		
≥70 years	73 (44.6)		
ASA at SM diagnosis, *n* (%)			
ASA ≤ 2	60 (36.1)		
ASA > 2	106 (63.9)		
Primarius, *n* (%)			
Lung cancer	41 (24.7)		
Breast cancer	22 (13.3)		
Prostate cancer	36 (21.7)		
Plasmacytoma	7 (4.2)		
GI cancer	10 (6)		
Kidney cancer	14 (8.4)		
Other pathologies	27 (16.3)		
CUP	9 (5.4)		
Ki-67 index, *n* (%)			
≤20%	102 (61.4)		
>20	64 (38.6)		
First manifestation as SM, *n* (%)			
Synchronous	72 (43.4)		
Metachronous	85 (51.2)		
SM locations, *n* (%)			
Craniocervical	1 (0.6)		
Cervical vertebrae	14 (8.4)		
Cervicothoracic	11 (6.6)		
Thoracic vertebrae	100 (60.2)		
Thoracolumbar	11 (6.6)		
Lumbar spine	24 (14.5)		
Lumbosacral	5 (3.0)		

ASA, American Society of Anesthesiology; ASIA, American Spinal Injury Association; CUP, cancer of unknown primary; GI, gastrointestinal; IQR, interquartile range; KPS, Karnofsky Performance Scale; SM, spinal metastases.

**Table 2:** Baseline clinical, neurological, imaging, and oncological characteristics of patients with a low (≤20%) and high (>20%) Ki-67 index. Bold *p*-values indicate statistically significant differences.

	Ki-67 ≤ 20%, *n* = 102	Ki-67 > 20%, *n* = 64	*p*-Value
**Patient demographics**			
Gender, *n* (%)			0.481
Male	63 (61.8)	36 (56.3)	
Female	39 (38.2)	28 (43.8)	
Age at SM diagnosis			0.768
Median (IQR)	68 (58.75–77.25)	66.5 (60–74)	
Range	51	59	
ASA at SM diagnosis, *n* (%)			**0.042**
ASA ≤ 2	43 (42.2)	17 (26.6)	
ASA > 2	59 (57.8)	47 (73.4)	
CCI, *n* (%)			0.445
<10	83 (81.4)	55 (85.9)	
≥10	19 (18.6)	9 (14.1)	
**Tumor characteristics**			
Primary, *n* (%)			0.074
Lung cancer	19 (18.6)	22 (34.4)	**0.022**
Breast cancer	17 (16.7)	5 (7.8)	0.102
Prostate cancer	24 (23.5)	12 (18.8)	0.467
Plasmacytoma	5 (4.9)	2 (3.1)	
GI cancer	4 (3.9)	6 (9.4)	0.077
Kidney cancer	12 (11.8)	2 (3.1)	0.051
Other pathologies	15 (14.7)	12 (18.8)	
CUP	6 (5.9)	3 (4.7)	
TTP, PT to SM diagnosis, years			**0.012**
Median (IQR)	1 (0–8)	0 (0–1.5)	
Range	30	12	
First manifestation as SM, *n* (%)			**0.048**
Synchronous	38 (39.6)	34 (55.7)	
Metachronous	58 (60.4)	27 (44.3)	
Previous treatment, *n* (%)			0.744
Yes	60 (58.8)	36 (56.3)	
No	42 (41.2)	28 (43.8)	
SM locations, *n* (%)			0.887
Craniocervical	0 (0)	1 (1.6)	
Cervical vertebrae	10 (9.8)	4 (6.3)	
Cervicothoracic	7 (6.9)	4 (6.3)	
Thoracic vertebrae	60 (58.8)	40 (62.5)	
Thoracolumbar	7 (6.9)	4 (6.3)	
Lumbar spine	15 (14.7)	9 (14.1)	
Lumbosacral	3 (2.9)	2 (3.1)	
Involved segments, *n* (%)			0.491
≤2 segments	55 (53.9)	31 (48.4)	
≥3 segments	47 (46.1)	33 (51.6)	
Spinal cord compression, *n* (%)			0.244
Yes	88 (86.3)	59 (92.2)	
No	14 (13.7)	5 (7.8)	
Spinal instability, *n* (%)			0.095
Yes	68 (70.1)	50 (82)	
No	29 (29.9)	11 (18)	
**Systemic disease burden**			
Extraspinal metastases, *n* (%)			0.119
Yes	62 (60.8)	31 (48.4)	
No	10 (39.2)	33 (51.6)	
Numbers of metastases sites			0.348
Median (IQR)	1 (0–2)	0.5 (0–2)	
Range	6	6	
**Preoperative functional and neurological status**			
Preoperative ASIA, *n* (%)			0.106
Good (D, E)	64 (62.7)	32 (50)	
Poor (A, B, C)	38 (37.3)	32 (50)	
Preoperative KPS, *n* (%)			0.104
≥70%	73 (71.6)	38 (59.4)	
<70%	29 (28.4)	26 (40.6)	
**Surgical treatment and oncological therapy**			
Surgery, *n* (%)			0.536
Only decompression	30 (29.4)	16 (25)	
Stabilization	72 (70.6)	48 (75)	
Operative time, minutes			0.462
Median (IQR)	179 (130.5–282.75)	195 (152.25–267.75)	
Range	551	413	
Intraoperative blood loss, mL			0.32
Median (IQR)	600 (350–1200)	600 (400–1000)	
Range	6950	2650	
**Postoperative outcomes**			
Specific SSCs, *n* (%)			0.57
Yes	16 (15.7)	8 (12.5)	
No	86 (84.3)	56 (87.5)	
Postoperative revision, *n* (%)			0.169
Yes	91 (89.2)	61 (95.3)	
No	11 (10.8)	3 (4.7)	
Postoperative ASIA, *n* (%)			0.223
Good (D, E)	73 (71.6)	40 (62.5)	
Poor (A, B, C)	29 (28.4)	24 (37.5)	
Postoperative KPS, *n* (%)			0.122
≥70%	68 (66.7)	35 (54.7)	
<70%	34 (33.3)	29 (45.3)	
Local tumor recurrence, *n* (%)			0.145
Yes	20 (22.5)	7 (12.7)	
No	69 (77.5)	48 (87.3)	
Readmission in 30 days, *n* (%)			0.295
Yes	7 (7.4)	2 (3.3)	
No	88 (92.6)	58 (96.7)	
Readmission in 3 months, *n* (%)			0.206
Yes	12 (13.3)	4 (6.8)	
No	78 (86.7)	55 (93.2)	
Overall survival, months			**0.001**
Median	14.5	5	
1-year mortality, *n* (%)			**0.001**
Yes	41 (41.8)	45 (78.9)	
No	57 (58.2)	12 (21.1)	

ASA, American Society of Anesthesiology; ASIA, American Spinal Injury Association; CCI, Charlson Comorbidity Index; CUP, cancer of unknown primary; GI, gastrointestinal; IQR, interquartile range; KPS, Karnofsky Performance Scale; PT, primary tumor; SM, spinal metastases; SSC, spinal surgery-related complication; TTP time to progression.

**Table 3:** Multivariate binary logistic regression of potential variables predicting a high Ki-67 index (>20%). *p*-values in bold represent statistically significant results.

Variable	aOR	95% CI	*p*-Value
Gender (female/male)	1.26	0.67–2.37	0.481
Age (≥70/<70 years)	0.835	0.432–1.613	0.591
ASA (>2/≤2)	2.133	1.053–4.32	**0.035**
CCL (≥10/<10)	0.646	0.269–1.555	0.33
BMI (normal/obesity)	0.836	0.304–2.301	0.729
Lung cancer as primary tumor (yes/no)	2.288	1.117–4.688	**0.024**
Breast cancer as primary tumor (yes/no)	0.348	0.118–1.032	0.057
Prostate cancer as primary tumor (yes/no)	0.592	0.262–1.34	0.208
GI cancer as primary tumor (yes/no)	2.072	0.560–7.621	0.273
Kidney cancer as primary tumor (yes/no)	0.197	0.042–0.937	**0.041**
Previously oncological treatment (yes/no)	0.9	0.478–1.693	0.744
First manifestation SM (syn/meta)	1.922	1.003–3.682	**0.049**
Thoracis vertebrae location (yes/no)	0.667	0.128–3.47	0.63
Involved segments (≥3/≤2 segments)	0.908	0.444–1.857	0.791
Spinal cord compression (yes/no)	1.287	0.408–4.055	0.667
Spinal instability (yes/no)	1.945	0.807–4.697	0.138
Extraspinal metastases (yes/no)	0.398	0.15–1.051	0.063
Surgery (stabilization/only decompression)	1.438	0.651–3.174	0.369
Postoperative systematic therapy (yes/no)	0.881	0.147–5.263	0.889
Postoperative radiotherapy (yes/no)	0.506	0.087–2.946	0.448
Specific SSCs (yes/no)	0.882	0.305–2.548	0.817
Postoperative revision (yes/no)	0.446	0.101–1.98	0.288
Preoperative ASIA (poor [A, B, C]/good [D, E])	1.609	0.673–3.846	0.285
Preoperative KPS (<70%/≥70%)	1.056	0.445–2.506	0.902
Postoperative ASIA (poor [A, B, C]/good [D, E])	1.124	0.463–2.728	0.797
Postoperative KPS (<70%/≥70%)	1.539	0.655–3.617	0.323

aOR, adjusted odds ratio; ASA, American Society of Anesthesiology; ASIA, American Spinal Injury Association; BMI, Body Mass Index; CI, confidence interval; KPS, Karnofsky Performance Scale; meta, metachronous; SM, spinal metastases; SSC, spinal surgery-related complication; syn, synchronous.

## Data Availability

The datasets generated and/or analyzed during the current study are available from the corresponding author on reasonable request.

## Supplementary Materials

The following supporting information can be downloaded at: https://www.mdpi.com/article/10.3390/cancers18081210/s1, Table S1. Multivariable Cox regression analysis of overall survival.

## Abbreviations

ASA	American Society of Anesthesiology
ASIA	American Spinal Injury Association
AUC	Area under the curve
CCI	Charlson comorbidity index
CUP	Cancer of unknown primary
CI	Confidence interval
GI	Gastrointestinal
IQR	Interquartile range
KPS	Karnofsky Performance Scale
Meta	Metachronous
N	Numbers
OR	Odds ratio
OS	Overall survival
PT	Primary tumor
ROC	Receiver operating characteristic
SD	Standard deviation
SINS	Spinal instability neoplastic score
SM	Spinal metastasis
SSCs	Spinal surgery-related complications
Syn	Synchronous
TTP	Time to progression
