# Supplementary material for: The Prognostic Impact of the Ki-67 Proliferation Index in Patients with Surgically Treated Spinal Metastases
Source: Cancers (Basel). 2026 Apr 10;18(8):1210. doi: 10.3390/cancers18081210 (PMC13114361; doi:10.3390/cancers18081210)
Supplement: Supplementary file 1 [file cancers-18-01210-s001.zip › cancers-4192750-supplementary.pdf]

Table S1. Multivariable Cox regression analysis of overall survival.

Variable	HR	95% CI	p-value
Ki-67 (> 20% / ≤ 20%)	1.981	1.318–2.977	0.001
Gender (female / male)	1.005	0.604–1.672	0.986
Age (≥ 70 / < 70 years)	0.966	0.628–1.484	0.874
Lung cancer as primary tumor (yes / no)	1.561	0.904–2.696	0.110
Breast cancer as primary tumor (yes / no)	0.575	0.287–1.15	0.118
Prostate cancer as primary tumor (yes / no)	0.767	0.431–1.365	0.367
Previous oncological treatment (yes / no)	1.916	1.123–3.269	0.017
First manifestation SM (syn / meta)	1.556	0.935–2.59	0.089
Involved segments (≥ 3 / ≤ 2)	1.078	0.661–1.76	0.763
Spinal cord compression (yes / no)	1.071	0.515–2.226	0.855
Spinal instability (yes / no)	0.629	0.329–1.204	0.162
Extraspinal metastases (yes / no)	1.308	0.867–1.972	0.200
Surgery (stabilization / only decompression)	1.521	0.789–2.933	0.210
Preoperative KPS (< 70% / ≥ 70%)	3.734	1.775–7.852	0.001
Postoperative KPS (< 70% / ≥ 70%)	0.474	0.214–1.052	0.066
Preoperative ASIA (poor [A, B, C] / good [D, E])	0.632	0.164–2.436	0.505
Postoperative ASIA (poor [A, B, C] / good [D, E])	2.447	0.574–10.435	0.227

ASIA, American Spinal Injury Association; HR, hazard ratio; KPS, Karnofsky Performance Scale; meta, metachronous; SM, spinal metastases; syn, synchronous.
